# Intraoperative Colonic Irrigation for Low Rectal Resections With Primary Anastomosis: A Fail-Safe Surgical Model

**DOI:** 10.3389/fsurg.2022.821827

**Published:** 2022-04-08

**Authors:** Jonas Herzberg, Shahram Khadem, Salman Yousuf Guraya, Tim Strate, Human Honarpisheh

**Affiliations:** ^1^Department of Surgery—Krankenhaus Reinbek St. Adolf-Stift, Reinbek, Germany; ^2^Department of Clinical Sciences, College of Medicine, University of Sharjah, Sharjah, United Arab Emirates

**Keywords:** rectal resection, anastomotic leakage, colonic irrigation, mechanical bowel preparation, rectal cancer

## Abstract

**Aim:**

Regardless the technological developments in surgery, the anastomotic leakage (AL) rate of low rectal anastomosis remains high. Though various perioperative protocols have been tested to reduce the risk for AL, there is no standard peri-operative management approach in rectal surgery. We aim to assess the short-term outcome of a multidisciplinary approach to reduce the rates of ALs using a fail-safe-model using preoperative and intraoperative colonic irrigation in low rectal resections with primary anastomosis.

**Methods:**

Between January 2015 and December 2020, 92 patients received low rectal resections for rectal cancer with primary anastomosis and diverting ileostomy. All these patients received pre-operative mechanical bowel preparation (MBP) without antibiotics as well as intraoperative colonic irrigation. The intraoperative colonic irrigation was performed *via* the efferent loop of the ileostomy. All data were analyzed by SPSS for descriptive and inferential analyses.

**Results:**

In the study period, 1.987 colorectal surgical procedures were performed. This study reports AL in 3 (3.3%) of 92 recruited patients. Other postoperative complications (Dindo-Clavien I-IV) were reported in 25 patients (27.2%), which occurred mainly due to non-surgical reasons such as renal dysfunction and sepsis. According to the fail-safe model, AL was treated by endoscopic or re-do surgery. The median postoperative length of hospitalization was 8 days (4–45) days.

**Conclusion:**

This study validates the effectiveness of a multi-disciplinary fail-safe model with a pre-operative MBP and an intraoperative colonic irrigation in reducing AL rates. Intraoperative colonic irrigation is a feasible approach that lowers the AL rates by reducing fecal load and by decontamination of the colon and anastomotic region. Our study does not recommend a pre-operative administration of oral antibiotics for colorectal decontamination.

## Introduction

Anastomotic leakage (AL) after colorectal surgery is a feared complication due to its high morbidity and mortality rates (1, 2). Though there is no consensus about a standard definition of AL, the “International Multispecialty Anastomotic Leak Global Improvement Exchange” has elaborated AL as “a defect of the integrity in a surgical join between two hollow viscera with communication between the intraluminal and extraluminal compartments” (3). In colorectal surgery, the reported incidence of ALs significantly varies according to the location of the anastomosis (4). Literature has reported a wide range of AL rates of 1 to 20% for all colonic locations; from 0 to 2% after colocolonic and 0.02 to 4% after enteroenteric and ileocolonic anastomoses (5). In the low rectal anastomoses, much higher ALs rates of up to 28% have been reported (6).

Besides the surgical volumes and surgeon's experience, which are decisive for surgical outcomes (7), additional factors such as mechanical bowel preparation (MBP) potentially influence short-term surgical outcomes and AL rates (8). Significant colonization of lower GI tract with aerobic and anaerobic microbes leads to infectious complications with resultant increased concentrations of collagenases and matrix metalloproteinases (9). This adversely affects stromal regeneration and leads to an early degradation of collagen at the anastomotic sites (10, 11). The purpose of MBP is to reduce the rate of surgical side infection (SSI) and AL by reducing fecal load and bacterial count in the colon (12). Using pre-operative oral antibiotics and MBP, the National Surgical Quality Improvement Program by the American College of Surgeons has shown an approximately 50% reduction of AL rates and superficial surgical site infections (SSIs) and better rates of 30-day mortality (13). Several other researchers have also endorsed the use of non-absorbable oral antibiotics and MBP in reducing the SSIs and ALs rates in colorectal surgery (14, 15).

Regrettably, controversy prevails about the impact of pre-operative MBP in colorectal surgery (16–18). In their multi-center randomized trial, Si-Oen et al. could not find significant difference in the outcome variables between patients with and without MBP in elective open colonic surgery (19). The authors argued that MPB may be discontinued in open colon surgery. Similarly, other researchers have discouraged the routine pre-operative use of MBP in colonic surgery (20, 21). In addition to the controversial role of MBP, some investigators have coined the possibility of intra-operative colonic irrigation for reducing AL rates in planned colorectal surgery (22, 23). The combination of pre-operative MBP and intra-operative colonic irrigation following a multidisciplinary approach may be an alternative that has not been rigorously investigated in the literature so far.

In our study, we aimed to evaluate the short-term outcomes after open and laparoscopic low rectal resections and primary anastomosis for rectal cancers using a multidisciplinary standardized fail-safe approach in colorectal surgery. This fail-safe approach, was first used in the engineering discipline and has now been widely adopted in the bioengineering field (24). According to this model, every potential error is secured by an additional safety net, so the magnitude of possible hazards is minimized. We adopted these safety nets for colorectal surgery including a wide range of pre-, peri- and postoperative steps. We measured surgical outcomes in terms of post-operative complications, particularly ALs, and report the effectiveness of the fail-safe model using pre-operative MBP and intraoperative colonic irrigation in rectal surgery.

## Methods and Material

### Patients' Cohort and Study Design

**Figure 1:**
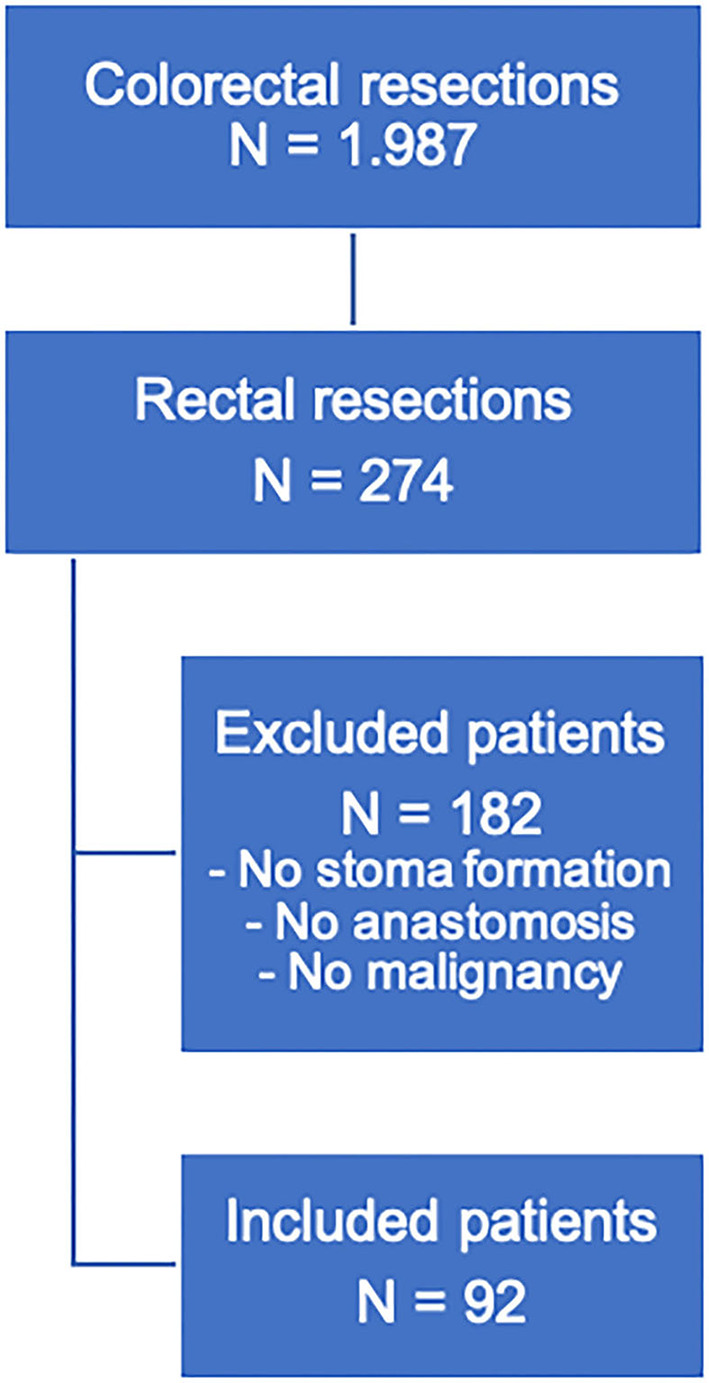
Flowchart for the selection of patients with rectal resections in this study. Patients with rectal resections for benign lesions, abdominoperineal extirpation, or no stoma formation (due to preoperative stoma or upper rectal resection) were excluded.

We recruited all consecutive patients with resectable rectal cancer undergoing elective surgical resections with primary anastomosis and protective ileostomy from January 2015 till December 2020 at Reinbek Hospital St. Adolf-Stift Germany (Figure 1). All patients were managed by a standard multidisciplinary approach of a fail-safe-mode as outlined in Table 1. We excluded all patients with benign rectal lesions, emergency rectal surgeries, patients with terminal stoma without anastomosis and patients without perioperative colonic irrigation or ileostomy. The patients with cancer of the middle (>6–12 cm from the anal verge) or lower third of the rectum (<6 cm from the anal verge) received a neo-adjuvant therapy if staged IIA (according to AJCC/UICC-classification) or higher (25) using a neoadjuvant chemoradiation following the multidisciplinary tumor board decision. Patients with lesions in the upper rectum (>12 cm from the anal verge) were included, if they were treated with low anterior resection due to tumor extension or if localized in the middle rectum preoperatively. We recorded the patients' demographics, body mass index (BMI), the American Society of Anesthesiologists (ASA) classification of physical health, tumor localization, open or laparoscopic surgical procedure, laparoscopic conversion to open surgery, length of hospital stay, complications according to Dindo-Clavien's classification (26) and 30-days-mortality.

**Table 1:** Fail-safe protocol for laparoscopic elective rectal resections in this study.

**Preoperative settings**	
Mechanical bowel preparation with 2l Endofalk^®^	**O**
Preoperative intravenous single-shot antibiotics	**O**
**Operative approach/technical aspects**	
Multidisciplinary team lead by an experienced colorectal surgeon	**O**
Complete mobilization of the hemicolon for tensions free anastomosis	**O**
Bleeding / perfusion test at the edge of resection margin	**O**
End-to-end anastomosis	**O**
– Mesentery is in one line with resection margin	**O**
– Do not free endings from fatty tissue	**O**
– Avoid sharp-angled edges	**O**
– Stretching of anal sphincter muscle for 3 minutes	**O**
– Spine of the stapling-device in direct contact with stapled line	**O**
– After joining ends, compression for at least 1 minute before release	**O**
– Anastomotic assessment using sigmoidoscope (air test + intraluminal inspection)	**O**
– Diverting stoma for low rectal anastomosis	**O**
– On-table-lavage over efferent loop of ileostomy with 5l of NaCl	**O**
– Place a drainage tube near the anastomosis	**O**
**Postoperative protocol**	
3 days liquid low-volume high-calorie nutrition (except patients with diverting stoma)	**O**
Full meals from 4th POD onwards	**O**
Endoscopic control of colorectal-/coloanal anastomosis on 4th POD	**O**
In case of insufficiency consideration of OTSC ^®^ application	**O**

MBP was performed one day before surgery using 2l of Endofalk^®^ (Dr. Falk Pharma GmbH^®^, Freiburg, Germany). No oral antibiotic was applied to the patients' cohort in our study. A peri-operative single shot antibiotic using 500mg metronidazole and 1,500 mg cefuroxime was given to all patients, half an hour before the incision, and was repeated at 4 h during surgery. The primary endpoint of the surgical therapy using the fail-safe model was the estimation of the rate of AL. We used endoscopy for the diagnosis of AL followed by a CT scan instead of a primary CT scan. The characteristics for AL were defined by the grading system proposed by Rahbari et al. (2). Postoperative morbidity was defined as complication occurring within 30 days after surgery, or during the same hospital stay.

### Surgical Procedure

**Figure 2:**
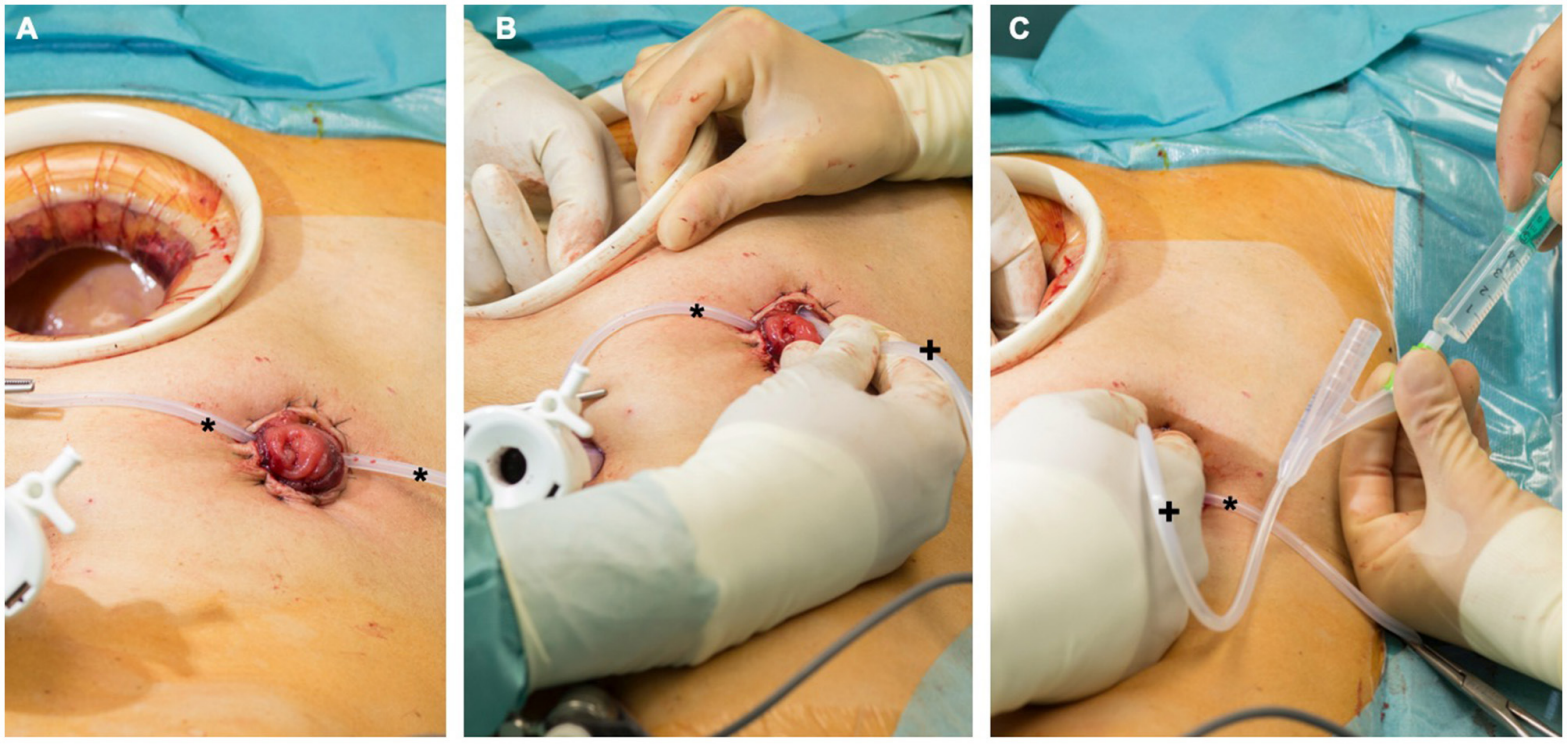
**(A)** Protective ileostomy after rectal resection. The Pfannenstiel incision is still protected by a wound retractor. A loop (*) is stabilizing the stoma during manipulation. **(B)** A urinary catheter (+) is placed in the efferent loop. **(C)** The catheter (+) is blocked under manual control with 5 ml before starting the antegrade colonic irrigation. The intestine can be checked within the procedure by one surgeon to prevent dislocation of the catheter or accidental perforation.

For rectal cancer resections with low rectal anastomosis, a full mobilization of the left hemicolon was routinely performed. During the laparoscopic rectal resection, first a nerve preserving total mesorectal excision (TME) was done. Then the dissection and resection of the rectum below the tumor about 1–3 cm from the anal verge with a linear stapler (45 mm EndoGIA™, Medtronic, Dublin, Ireland) was performed. In case of large specimen, additional stapling catridges were used. In case of an open procedure, the transection of the rectal specimen was done using an Echelon CONTOUR™ device (Ethicon, Raritan USA). The proximal division was performed extracorporially through a Pfannenstiel incision. Before performing the anastomosis, the sphincter muscle was manually stretched. The anastomosis was performed intracorporially using a transanally introduced circular stapling device (28 mm circular stapling device, Metronic, Dubin, Ireland) with the spine in contact to the linear stapling line (27). Before performing the anastomosis, a compression for at least 60 s was done to reduce the tissue edema. An air-leakage test was routinely performed afterwards. A protective ileostomy was conducted for all low rectal anastomosis at the terminal ileum loop. An additional intraoperative colonic irrigation was installed with 5 liters of warm saline *via* efferent loop of the ileostomy. For this procedure, a urinary catheter was inserted into the efferent loop that was blocked by manual control with 5 ml of sterile water to prevent a massive retrograde discharge (Figure 2). To secure the anastomosis, a second surgeon would manually stretch the anal orifice to ensure a seamless outflow. The outflow was visually examined for persisting fecal load by the second surgeon and the procedure was continued until the outflow was clear and without any visible fecal load. Afterwards, a soft drainage tube was placed intracorporeally near the site of anastomosis (Table 1). On the 4th postoperative day, an endoscopy was performed to confirm the anastomotic integrity and then the soft drainage tube was removed.

AL was defined as “a defect of the intestinal wall integrity at the ileocolic, colorectal or coloanal anastomotic site (including suture and staple lines of neorectal reservoirs) leading to a communication between the intra- and extraluminal compartments” (2). A pelvic abscess close to the anastomosis was also considered as anastomotic leakage (2). AL was graded according to the standard classification into grade A, B or C.

### Statistics and Ethics

Statistical analyses were performed using IBM SPSS Statistics Version 25 (IBM Co., Armonk, NY, USA). All variables were listed as means with standard deviation. Categorical variables were arranged as numbers with percentages.

This study was conducted in accordance with the declaration of Helsinki (28). Ethical approval was waived by the local Ethics Committee of the Medical Association Schleswig-Holstein as this is a retrospective study and all the procedures being performed were part of the routine care.

## Results

Between January 2015 and December 2020 1,987 colorectal surgical procedures were performed in the study center. This included a total of 274 (13.8%) rectal resections including Hartmann procedures or abdominoperineal extirpations. 92 patients were treated for rectal cancer and underwent therefor low rectal resections with primary anastomosis and protective ileostomy using the fail-safe model including an intraoperative colonic irrigation.

Of the 92 patients, 61 (66.3%) were men and 31 (33.7%) were women, with a mean overall age of 64.40 years (range 37–86 years). In 88 (95.7%) patients, a laparoscopic approach was used, while four patients were treated by laparotomies (4.3%). The patients' characteristics in this study are listed in Table 2.

**Table 2:** Characteristics of the study cohort (*n* = 92).

**Age**, years (mean ± SD)	64.4 ± 11.66
**Body Mass Index** (mean ± SD)	27.15 ± 4.84
**Sex (%)**	
Male	61 (66.3)
Female	31 (33.7)
**UICC/AJCC (%)**	
0	5 (5.4)
I	36 (37.1)
II	17 (18.5)
IIIA	13 (14.4)
IIIB	12 (13.1)
IIIC	4 (4.3)
IV	5 (5.4)
**ASA classification (%)**	
ASA 1	4 (4.3)
ASA 2	69 (75.0)
ASA 3	19 (20.7)
ASA 4	0 (0)
**Tumor localization (%)**	
Lower rectum (<6 cm)	34 (37.0)
Middle rectum (6–12 cm)	47 (51.1)
Upper rectum (12–16 cm)	11 (12.0)
**Approach (%)**	
Open	4 (4.3)
Laparoscopic	88 (95.7)
Number of used stapling devices, mean (Range)	2.3 (1–4)
Neoadjuvant treatment (%)	47 (51.1)
**Comorbidity (%)**	
Arterial Hypertension	43 (46.7)
Smoking	11 (12.0)
Diabetes mellitus	12 (12.0)

An anastomotic leakage occurred in 3 (3.3%) cases. Two case of type B rectal insufficiency according to the classification by Rahbari et al. were diagnosed via endoscopic assessment, and they were treated by endoscopic vacuum therapy. One patient needed a re-operation due to an extended wall deficit.

Post-operative complications were reported in 25 (27.2%) patients that were grouped according to the Dindo-Clavien's classification; 7 (7.6%) grade I, 6 (6.5%) grade II, 0 (0%) grade IIIa, 7 (7.6%) grade IIIb and 5 (5.4%) grade IV cases were reported. There was no mortality during hospital stay and within first 30 days after surgery. Table 3 provides an overview of the short-term postoperative outcomes and complications. Of the cases with grade IIIb complications, one patient had prolonged paralysis, two cases had postoperative subcutaneous hematoma, which needed evacuation, two AL treated by endoscopy, one by re-operation and one perioperative perforation of the ileum. The median postoperative length of hospitalization was 8 (4–45) days.

**Table 3:** Outcome after intraoperative colonic irrigation (*n* = 92).

**Outcome**	***n*** **(%)**
Anastomotic leakage	3 (3.3)
Prolonged paralysis	5 (7.1)
Kidney failure	4 (5.7)
Pneumonia	2 (2.9)
Surgical side infection	1 (1.4)
Other	8 (11.4)
Postoperative bleeding	2 (2.2)
Length of hospital stay after surgery [days] (mean ± SD)	10 ± 6.55

*Some postoperative complications occurs in the same patient*.

*SD, standard deviation*.

## Discussion

In our study, using a standardized fail-safe approach including a pre-operative MBP and peri-operative colonic irrigation, we report an over-all complication rate of 27.2% with AL rate of 3.3%. The fail-safe approach includes pre- and intra-operative colonic irrigation as a core component of the multi-step peri-operative management plan for low rectal resections.

The use of colonic irrigation before and during surgery provides a foundation for a safe anastomosis by reducing intracolonic pressure, fecal load, and bacterial count in the vicinity of anastomosis (29, 30).

### Preoperative Mechanical Bowel Preparation

As first reported by Nichols and Condon in 1971, MBP is associated with a reduced complication rate following colorectal surgery (31). In contrast, some large data sets have shown that pre-operative MBP alone has no influence on post-operative complications such as SSI or AL (16, 32, 33). However, the combination of non-absorbable oral antibiotics with pre-operative MBP was shown to be beneficial in preventing and reducing SSI and AL (18, 34, 35). Currently, this combination of pre-operative antibiotics and MBP is frequently used worldwide with success. A recently published large retrospective registry study including more than 20,000 patients showed a significantly lower SSI and AL rates after combined MPB with oral antibiotics, whereas the research did not report benefit of MBP when used alone (36).

MBP has not been widely adopted by the European colorectal surgeons. The reasons for this reluctance are multifactorial, but the trend toward enhanced recovery after surgery (ERAS) protocols that excludes routine MBP is probably a significant contributor (37). According to the fail-safe model used in our study and, in contrast to some randomized controlled trials, we used MPB without oral antibiotics but with an intra-operative colonic irrigation in rectal surgery. This approach resulted in lower AL rates than those reported by Klinger et al. (36). In their study on a total of 27,804 patients, 5,471 patients underwent surgery without pre-operative preparation, 7,617 received MBP alone, 1,374 were given antibiotic bowel preparation (ABP) alone, while 8,885 patients received both ABP and MBP. The patients with dual preparation showed less rates of SSIs and ALs (OR = 0.53, *p* < 0.001). The study has recommended a routine use of ABP and MBP in elective colorectal resections. In contrast, we used MBP and peri-operative colonic irrigation with even better results. In 2017, Ji et al. have a large single-center data on more than 1,300 rectal cancer resections. The authors have shown that AL rate did not significantly differ with or without MPB but remained substantially high with 7.81% vs. 9.27%, respectively (38). Nevertheless, until recently, the published data has shown AL rates of higher than 5% regardless of ABP or MBP alone or in combination. Of course, our data with a leakage rate of 3.3% comes from a retrospective single center cohort study and has to be carefully compared with the results of randomized controlled trials mentioned earlier. Nevertheless, an AL-rate below 5% in rectal cancer surgery is promising and needs further evaluation.

### Intraoperative Colonic Irrigation

Even after meticulous pre-operative bowel preparation, the colon is usually not completely mechanically cleaned and fecal particles and ingested roughage are still left in the colon. In our study, beside pre-operative bowel preparation, diverting ileostomy and intra-operative colonic irrigation were performed *via* efferent loop of the ileostomy. These two additional measures were taken in order to decontaminate the colon and thus mitigating the risk of AL. Intra-operative colonic irrigation was first introduced by Muir et al. (39), and was modified by Dudley and co-workers proposing antegrade on-table colonic irrigation with primary anastomosis (22, 29). Interestingly, various authors have argued that intraoperative colonic irrigation with primary anastomosis was feasible for left sided resections (40–42). The intra-operative colonic preparation would be more valuable in unprepared or inadequately prepared bowels in emergency situations and in tumorous stenosis. There is also enthusiasm for the on-table colonic irrigation with an additional on-table colonoscopy especially when a pre-operative colonoscopy is not feasible due to emergency or tumor stenosis (42).

Several studies have shown that performing colonic irrigation intraoperatively can potentially reduce the rate of Hartman's procedures (22, 43). However, there is no reported data that can establish the effectiveness of routine pre-operative MBP in combination with on-table colonic irrigation as demonstrated by the fail-safe model in our study. Such approach offers another opportunity of cleansing the colon as well as the rectal anastomosis for better oncological surgical outcomes.

### Fail-Safe-Protocol

The key elements of our fail-safe model for lower rectal resections include MBP, intraoperative colonic irrigation with drainage near anastomosis, proximal ileostomy and a routinely performed endoscopic assessment on the 4th postoperative day. Using this protocol, our study showed a low rate of AL (3.3%). Such encouraging results are often attributed to the surgeon's experience, which is truly vital. However, over a span of more than 5 years and in the presence of different operating surgeons with various levels of experience in a teaching hospital, higher complication rates could be expected. Following our fail-safe-protocol, standardized steps are elaborated not only for pre- and post-operative course but also during surgery. Especially in the phase of reconstruction the elaborated steps are clearly defined. This means a routinely perfusion test exactly at the resection margin and the preservation of fatty tissue from one or the other end to reduce perfusion deficiency. The anastomosis in the rectal resections were routinely performed using a circular stapling devices and end-to-end reconstruction. Before performing the anastomosis, a routinely stretching of the sphincter muscle was done. Then, the spine of the device would pierce in direct contact to the stapling line and a slow close approximation was followed by a compression for at least 1 min. This reduces tissue edema to ensure a safe staple-line. The functional outcomes after reconstruction in rectal surgery is a key element and the German Guidelines of Colorectal Cancer favor a non-straight anastomosis, as this strategy has shown better functional results, especially in the early postoperative period (44, 45). A retrospective analysis of the postoperative functional outcome following the fail-safe-approach showed a reduced AL rate without adverse functional outcomes or quality of life (27).

From a different perspective, intraoperative colonic irrigation might be beneficial if an AL occurs because of the reduced fecal load. Historically, the treatment of choice for a leaking colorectal or coloanal anastomosis had been a resection of the anastomosis followed by a Hartmann's procedure. Pelvic abscesses are often drained percutaneously under a CT-guided approach. Our study demonstrates that the incidence of pelvic abscess or peritonitis and especially the scale of complications resulting from AL can be avoided by intraoperative colonic irrigation integrated into a multidisciplinary fail-safe protocol.

### Study Limitations

Our study results are drawn from a small sample size in a single center setting with a heterogenous study cohort. Due to several reasons, including explicit inclusion criteria for rectal cancers, not all patients with rectal surgery could be included in this analysis. In addition, the retrospective design of this study indicates possible selection bias. Lastly, an absence of a control group due to its retrospective design did not allow us to report a case-control study. Though our results are promising, larger clinical trials in multi-center settings using a randomization are needed to help establish the effectiveness of our fail-safe model including the described intraoperative colonic irrigation.

## Conclusion

This study concludes that a low rate of AL in elective low rectal resections is feasible. This can be achieved by adopting a standardized fail-safe model peri-operative protocol. In the study center, this includes a pre-operative MBP, an intra-operative colonic irrigation to reduce fecal load at anastomotic site, a covering protective ileostomy and endoscopic evaluation on the 4th postoperative day. Even not all the peri-operative steps are evidence based, the presented AL rate is promising. A low rate of AL potentially reduces the concomitant complications of pelvic abscess, peritonitis, paralytic ileus and SSIs. As this is a retrospective cohort study reporting a single-center experience, further studies are essential, especially including emergency and training procedures, that can potentially validate our fail-safe-model using intra-operative colonic irrigation.

## Data Availability Statement

The original contributions presented in the study are included in the article, further inquiries can be directed to the corresponding author.

## Ethics Statement

Ethical review and approval was not required for the study on human participants in accordance with the local legislation and institutional requirements. Written informed consent for participation was not required for this study in accordance with the national legislation and the institutional requirements.

## Author Contributions

JH collected and analyzed the data and drafted the manuscript. SK developed the fail-safe-concept and drafted the manuscript. SG performed a scientific enrichment, linguistics manuscript drafting, and statistical analysis. TS reviewed the manuscript and supervised the implementation of the new concept. HH reviewed the manuscript and made an impact on discussion. All authors read and approved the final manuscript.

## Conflict of Interest

The authors declare that the research was conducted in the absence of any commercial or financial relationships that could be construed as a potential conflict of interest.

## Publisher's Note

All claims expressed in this article are solely those of the authors and do not necessarily represent those of their affiliated organizations, or those of the publisher, the editors and the reviewers. Any product that may be evaluated in this article, or claim that may be made by its manufacturer, is not guaranteed or endorsed by the publisher.
